# Aortoesophageal Fistula Occurring Due to Aortic Aneurysm

**DOI:** 10.7759/cureus.42148

**Published:** 2023-07-19

**Authors:** Snehasis Das, Sagar Prakash, Shweta Singh, Oseen Shaikh, Gopal Balasubramanian

**Affiliations:** 1 Surgery, Jawaharlal Institute of Postgraduate Medical Education and Research, Puducherry, IND; 2 Radiodiagnosis, Jawaharlal Institute of Postgraduate Medical Education and Research, Puducherry, IND

**Keywords:** atherosclerosis, aortoenteric fistula, hematemesis, aortic aneurysm, aortoesophageal fistula

## Abstract

Upper gastrointestinal bleeding is a rare presentation of the aortoesophageal fistula (AEF) and is usually caused by thoracic aortic aneurysms. We present the case of a 61-year-old male who presented with chest pain and hematemesis. A chest X-ray showed a widened mediastinum. The patient underwent computed tomography angiography (CTA), which showed the presence of a large aneurysm in the aorta, which caused compression of the trachea, esophagus, and left pulmonary artery. Additionally, there was evidence of an AEF. It was decided to perform an emergency surgical intervention on the patient. However, the patient had multiple episodes of hematemesis and expired.

## Introduction

Aortoesophageal fistula (AEF) is a rare cause of upper gastrointestinal bleeding, but it is often fatal if not treated early. An abnormal connection between the aorta and the esophagus causes it. AEF can be primary or secondary. Primary AEF occurs from aortic aneurysm and secondary due to malignancy or aortic graft repair [1]. Patients usually present with the classic Chiari's triad characterized by chest pain, a sentinel episode of hematemesis followed by a massive episode of hematemesis. Diagnoses are usually made by upper gastrointestinal endoscopy (UGIE) and computed tomography. This condition has a nearly 100% mortality rate without treatment and a 75% mortality rate with surgical intervention [1,2]. It is imperative to diagnose and treat early to prevent decompensation. We report the case of a 61-year-old male patient who experienced upper gastrointestinal bleeding and was diagnosed with AEF. Unfortunately, the patient's condition deteriorated due to recurrent episodes of hematemesis, ultimately resulting in his death.

## Case presentation

A 61-year-old male presented with complaints of blood in vomitus for three days and retrosternal chest pain radiating to the back for five days. He also had one episode of generalized tonic-clonic seizure in the preceding period, which was managed symptomatically at an outside center before being referred to our center for further management. He was a chronic alcoholic and smoker for 20 years. He was diagnosed with diabetes mellitus and hypertension 10 years ago and was on irregular medications.

**Figure 1:**
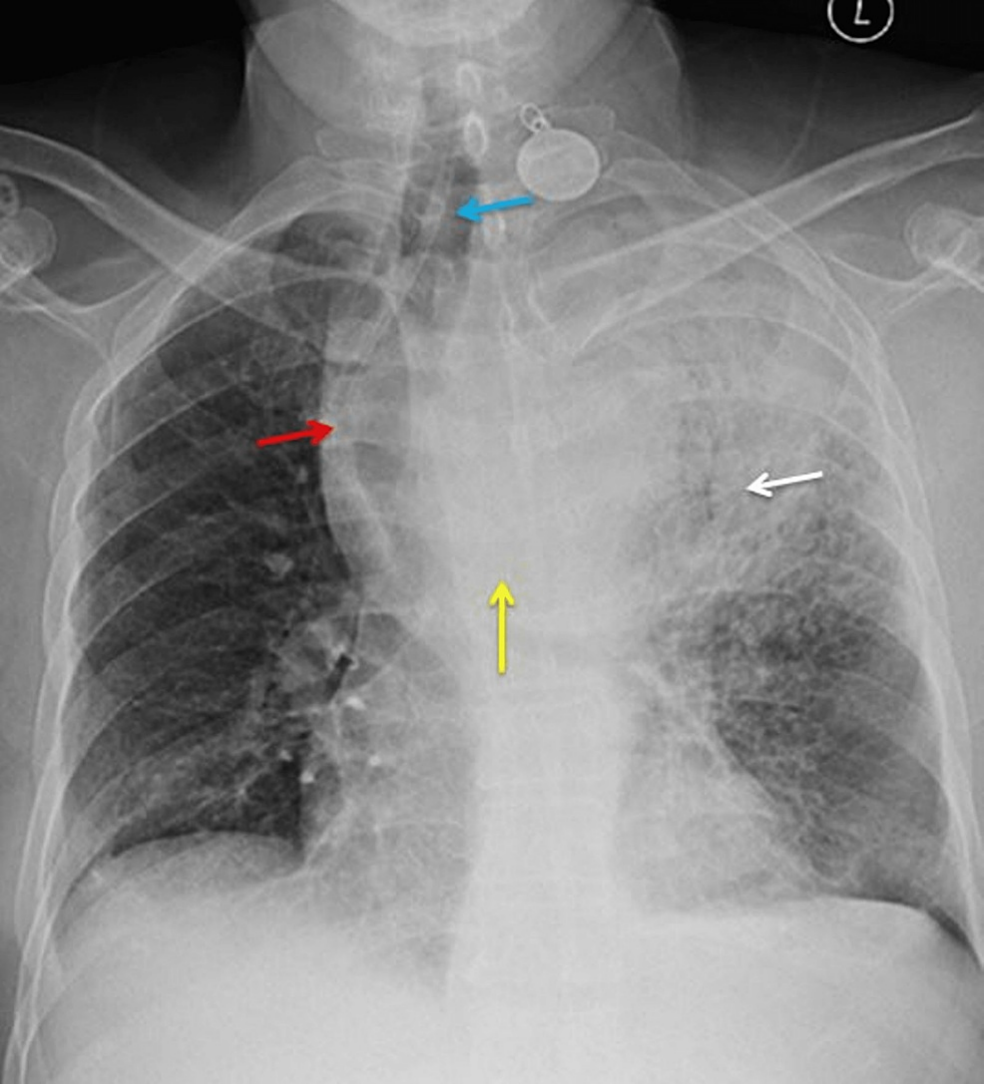
Chest X-ray showing haziness in the left lung (white arrow), aneurysm (yellow arrow, right-side shift of the trachea (blue arrow), widened mediastinum, and right-sided shift of the esophagus containing Ryle’s tube (red arrow)

Routine blood investigations showed severe anemia with a hemoglobin of 5 g/dl and mild leucocytosis. The other blood investigations, including renal and liver function tests, were normal. A chest X-ray revealed a tracheal deviation to the right side with significant opacity of the upper and middle zones of the left lung. There was also evidence of widened mediastinum and a shift of the esophagus toward the right side (Figure 1).

**Figure 2:**
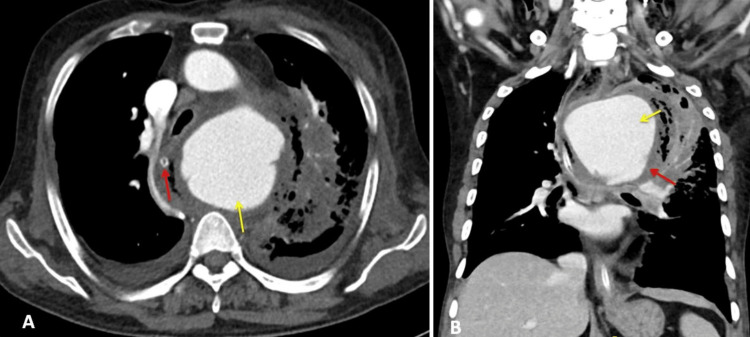
Computed tomography of the thorax. A (axial view): Aortic aneurysm (yellow arrow) and right side shift of the esophagus containing Ryle’s tube (red arrow). B (coronal view): Aortic aneurysm (yellow arrow) and eccentric thrombus (red arrow)

The patient underwent computed tomography angiography (CTA), which showed a lobulated saccular aneurysm with an eccentric thrombus within the aneurysm arising from the aortic arch distal to the origin of the left common carotid artery. The length of the aneurysm is 6.2 cm, and the width is 8.4 cm, extending from the T3 vertebral level to the T7 vertebral level. This aneurysm displaces the trachea and esophagus to the right side (Figure 2).

**Figure 3:**
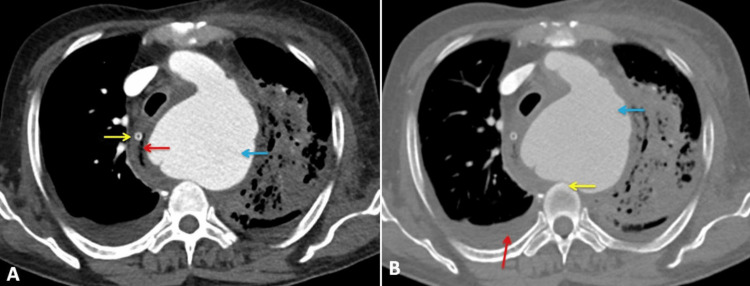
Computed tomography of the thorax. A (axial view): Aortic aneurysm (blue arrow), right side shift of the esophagus containing Ryle’s tube (yellow arrow), and probable site of fistula formation between aneurysm and esophagus. B (axial view, bone window): Aortic aneurysm (blue arrow), contact of the aneurysm with the vertebral body (yellow arrow), and pleural effusion (red arrow)

The posterior wall of the aneurysm is flat and touches the vertebral bodies. The left subclavian artery arises from the aneurysm. The aneurysm appears to compress the left main pulmonary artery as well. There was also evidence of bilateral moderate pleural effusion (Figure 3).

**Figure 4:**
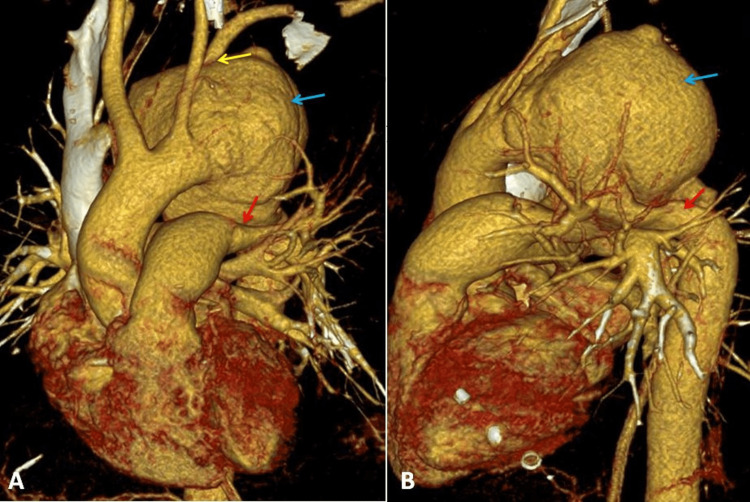
Three-dimensional reconstructed computed tomography image of the thorax. A (anterior view): Aortic aneurysm (blue arrow), the origin of the left subclavian artery from the aneurysmal sac (yellow arrow), and compression of the left main pulmonary artery (red arrow). B (lateral view): Aortic aneurysm (blue arrow) and compression of the aorta (red arrow)

We tried to reconstruct the three-dimensional picture of the computed tomography image of the aneurysm for better delineation and understanding (Figure 4).

He was scheduled for emergency surgical intervention by endovascular stenting and admitted to the intensive care unit for initial resuscitation. However, the patient had repeated episodes of hematemesis and could not recover despite the best resuscitative efforts.

## Discussion

AEFs are rare and fatal. It is paramount that patients receive an early diagnosis and prompt surgical intervention to survive. AEF can be classified as primary or secondary. When there is direct communication between the aneurysm and the adjacent bowel, it is called a primary AEF. Atherosclerotic aortic aneurysms most commonly cause this. Few are diagnosed when imaging is done for other causes, during surgery, or an autopsy [2]. Secondary AEFs occur after the endovascular or surgical repair of aortic aneurysm (80%). This can also occur due to the foreign body causing perforation, esophageal malignancy, and post-graft repair of the aorta as a postoperative complication [1,3]. The common sites of AEFs are the duodenum, esophagus, and small bowel. Our patient developed AEF, which was probably due to the presence of a thoracic aortic aneurysm. This stemmed from an underlying atherosclerosis.

Chiari describes AEF symptoms as a triad [4,5]. The triad consists of mid-thoracic pain, a sentinel episode of hematemesis, followed by massive hematemesis [5,6]. The diagnosis of AEF is often missed when the patient presents in the symptom-free interval or is confused for other benign causes like Mallory-Weiss tear when the patient improves after a period of fresh blood vomiting. Herald hemorrhages are brief, minor, and self-limiting bleeding events due to mucosal ulceration and localized necrosis rather than a real aortoenteric connection [7]. The actual hemorrhage and the warning period might range between hours and months. Patients with AEF also present similar to other AEF, with gastrointestinal bleeding either with hematemesis or haematochezia. They also have chest pain, breathlessness, or sepsis. Our patient presented with Chairi’s triad as described before and died before intervention was done.

A chest X-ray may be the initial investigation performed on these patients; however, it cannot be used as a screening tool. It may show the presence of a widened mediastinum, abnormal aortic contour, size, or mural calcification. In our case, there was a widened mediastinum with a shift of the trachea ad esophagus to the right side. UGIE is usually the first investigation done in stable patients with upper gastrointestinal bleeding [7]. It may or may not show evidence of the AEF. A bleeding ulcer or ulcer covered in blood clots should, however, be suspicious. Although we had planned UGIE for our patient, the patient became unstable with repeated vomiting.

CTA is the preferred diagnostic method for evaluating AEF [7]. In patients with AEF, CTA may show a leak of the contrast gastrointestinal tract, suggesting a fistula. Some other signs can be seen in patients with AEF, including air foci around the aorta or within the lumen of the aorta. There can be thickening of the bowel wall near the aneurysm, hematoma within the bowel wall, lumen, or mediastinum. An obliterated fat plane will be between the aneurysm and the bowel wall. However, the drawback is that when the patient is symptom-free, the aortogram may be normal due to transient clot formation [6-9]. In our case, CTA revealed an aneurysm from the arch of the aorta just distal to the origin of the left common carotid artery. However, there was no contrast extravasation directly into the esophagus from the aorta, which could have been probably due to the eccentric thrombus covering the fistulous site. Transthoracic echocardiography can diagnose thoracic aortic aneurysm except for the small part of the ascending aortic aneurysm. TTE cannot visualize this and needs transesophageal echocardiography for visualization [10]. MRI is used in patients when there is a contraindication for CTA [11-13].

After initial resuscitation, AEF needs immediate surgical intervention to be repaired. Without immediate operative repair, AEF is considered 100% fatal [1]. Surgery is the mainstay of AEF therapy. The surgical approach to primary and secondary AEF is the same. Extra-anatomic bypass with aortic ligation or in-situ reconstruction can be done. The in-situ reconstructions are of three types [10]. The AEF is usually treated by simple closure, resection with prosthetic graft placement, or bypass grafting. For individuals considered unstable or unsuitable candidates for surgical intervention, the placement of endovascular stent grafts may serve as a potential alternative. This may be a bridging therapy or a definitive treatment option [3]. However, approaches to preserving arterial perfusion have steadily gained popularity due to the poor results of aortic stump ligation and axilla-bifemoral bypass in situ. Small fistulas are usually treated by primary closure. Our patient was planned for emergency endovascular intervention but died due to multiple episodes of hematemesis.

## Conclusions

AEF is an uncommon cause of gastrointestinal bleeding that can be deadly if not recognized and treated correctly. Many patients may present with chest pain and small hematemesis and deteriorate later. If the patient has significant hematemesis, decompensated shock, or septicemia, the treating surgeon should be suspicious of AEF, especially in older patients. Because UGIE has a low diagnosis rate, CTA is the first-line imaging modality for identifying AEF. Echocardiography will also be helpful. As a result, coordinated management with emergency intervention plays a key role.
